# Determinants of compulsory hospitalisation at admission and in the course of inpatient treatment in people with mental disorders—a retrospective analysis of health records of the four psychiatric hospitals of the city of Cologne

**DOI:** 10.1186/s12888-022-04107-7

**Published:** 2022-07-14

**Authors:** Sönke Johann Peters, Mario Schmitz-Buhl, Olaf Karasch, Jürgen Zielasek, Euphrosyne Gouzoulis-Mayfrank

**Affiliations:** 1 LVR Institute for Healthcare Research, Wilhelm-Griesinger-Strasse 23, 51109 Cologne, Germany; 2 University Hospital of Cologne, Cologne, Germany; 3 LVR Clinics Cologne, Wilhelm-Griesinger-Strasse 23, 51109 Cologne, Germany; 4 Medical Faculty, Heinrich Heine University Düsseldorf, Düsseldorf, Germany

**Keywords:** Mental Health Act, Involuntary admission, Machine learning, Random Forest, CHAID

## Abstract

**Background:**

We aimed to identify differences in predictors of involuntary psychiatric hospitalisation depending on whether the inpatient stay was involuntary right from the beginning since admission or changed from voluntary to involuntary in the course of in-patient treatment.

**Methods:**

We conducted an analysis of 1,773 mental health records of all cases treated under the Mental Health Act in the city of Cologne in the year 2011. 79.4% cases were admitted involuntarily and 20.6% were initially admitted on their own will and were detained later during the course of in-patient stay. We compared the clinical, sociodemographic, socioeconomic and environmental socioeconomic data (ESED) of the two groups. Finally, we employed two different machine learning decision-tree algorithms, Chi-squared Automatic Interaction Detection (CHAID) and Random Forest.

**Results:**

Most of the investigated variables did not differ and those with significant differences showed consistently low effect sizes. In the CHAID analysis, the first node split was determined by the hospital the patient was treated at. The diagnosis of a psychotic disorder, an affective disorder, age, and previous outpatient treatment as well as the purchasing power per 100 inhabitants in the living area of the patients also played a role in the model. In the Random Forest, age and the treating hospital had the highest impact on the accuracy and decrease in Gini of the model. However, both models achieved a poor balanced accuracy. Overall, the decision-tree analyses did not yield a solid, causally interpretable prediction model.

**Conclusion:**

Cases with detention at admission and cases with detention in the course of in-patient treatment were largely similar in respect to the investigated variables. Our findings give no indication for possible differential preventive measures against coercion for the two subgroups. There is no need or rationale to differentiate the two subgroups in future studies.

## Background

All European countries and many in the world have mental health laws that allow involuntary admission to psychiatric hospitals in cases of an acute illness bearing considerable risk of self-harm or harm to others [1–3]. Cases of admission under state mental health laws are rising during the last years, and therefore it is particularly important to understand the reasons for these developments in order to plan targeted preventive measures [4]. A recent meta-analysis by Walker et al. [5] determined patient-related, systemic and environment-related risk factors for involuntary hospitalisation. Patients were more likely to be detained when being unemployed, male, single or previously married, when being diagnosed with psychotic or bipolar affective disorders and when having had a history of previous involuntary admissions. On a systemic level, previous contact to a general practitioner, social support and availability of home visits were identified as protective factors, whereas the involvement of police and a high level of area deprivation such as higher rates of unemployment, increased population density and less homogeneity of incomes in the living area of the patient qualified as risk factors [5].

In the literature on determinants and risk factors for involuntary psychiatric inpatient treatment the term *involuntary admission* is used in multiple different ways. When inspecting the more recent studies included in the meta-analysis by Walker et al. [5], in most studies it is not indicated whether or not involuntary *admissions* also include cases that switched from voluntary to involuntary legal status in the course of treatment [6–32]. It remains unclear whether in these cases later involuntary hospitalisation was treated as voluntary or as involuntary, or whether these cases were excluded from analysis. In some studies with a longer time of observation, the status of *voluntary* or *involuntary* was defined by one among several admissions [34–37]. Some studies defined the admission status as involuntary if there had been at least one involuntary admission of the respective patient in the entire time of observation [33–35]. Another study defined the legal status based on the first time the respective patient had been hospitalised during the observation period irrespective of the legal status of subsequent hospital stays [36]. Only few studies specified explicitly whether or not cases with switched from *voluntary* to *involuntary* legal status were included [37, 38] or excluded from the analysis [39].

It is unclear, whether it is helpful or necessary to differentiate between the two subgroups and study them separately. Differences between the two subgroups might be potentially relevant when planning targeted preventive measures. Hence, if the two subgroups were different in respect to risk factors, it might be possible to plan different tailored interventions against coercion. To our knowledge, the potential differences of these two subgroups have never been investigated. This was the goal of this study in which we conducted a secondary analysis of data from a previous publication [38].

## Methods

### Setting

With its about one million inhabitants, Cologne is Germany’s fourth largest city. At the time of the present study inpatient psychiatric care in Cologne was provided by four hospitals. Each of them provided care to the population of a certain geographical sector of the city ranging from approximately 100,000 to 500,000 inhabitants. The Mental Health Act of the federal state of North Rhine Westphalia (PsychKG NRW) allows involuntary hospitalisation for individuals who are mentally ill if they present an immediate, severe threat to themselves or others and will not agree to be hospitalised. The PsychKG requires “a physician with experience in the field of psychiatry” to activate the relevant sections of the Mental Health Act with a report to the responsible municipal authority explaining the need for immediate confinement. On the same day or latest the day after admission, a court hearing must be held which decides about detention. One single Municipal court is responsible for all involuntary hospitalisations in Cologne.

### Data sources and study design

In this secondary analysis we used the data of a previous retrospective study which analysed the health records of 5,764 inpatient cases of the four psychiatric hospitals in Cologne in the year 2011 [38]. The study included data of all 1,773 cases who were treated under the PsychKG NRW (Mental Health Act) and 3,991 cases who were treated voluntarily in the same hospitals and the same period of time (random sample out of 8,398 voluntary cases). Administrative, clinical, sociodemographic and socioeconomic data were extracted from the health records of each individual case by five trained assistant physicians. Diagnoses according to WHO ICD-10 classification [40] included both the main and all secondary diagnoses. The individual datasets were enriched by environmental socioeconomic data (ESED) characterizing the living environment of each case. The ESED were obtained from RWI-GEO-GRID [41] and provided information on household structures, house types, employment and unemployment as well as purchasing power for 1 × 1 km small grid cells [42]. The ESED and individual case data were merged through the postal code of the patient’s home address. More background information and details on the collection of the dataset can be found in our previous publications [38, 43].

In the present analysis we divided the 1,773 cases who were treated under the PsychKG NRW into two groups: Cases who were admitted to a psychiatric hospital against their will (*n* = 1,367) and cases who were admitted initially on their own will and were confined according to the Mental Health Act at some later point during in-patient treatment (*n* = 352). *n* = 54 cases were detained under legal guardianship (Civil Law Code/ BGB § 1906) at admission, but they also had a PsychKG issued later during their treatment. These cases were excluded from further analysis. All cases were anonymised and therefore, one patient can be represented with more than one case if having been admitted multiple times in 2011. Subsequently, the term *cases* instead of *patients* is used in this study.

### Statistical analysis

For categorical data such as diagnoses, most sociodemographic data and other clinical details, chi-square tests were used and Bonferroni-adjusted. Both the main and secondary diagnoses were analysed. Separately, the comorbidities addiction and psychosis (F1 and F2), as well as addiction and personality disorder (F1 and F6) were investigated, as they are known to correlate with higher rates of aggressive behaviour, violent crimes, impulsivity and self-harming behaviour [44–47]. Metric data such as age, length of inpatient stay and the environmental sociodemographic characteristics were analysed by means of Mann–Whitney-U-Tests. The level of significance was set at *p* ≤ 0.05. For significant differences, we used Cramér’s V and Cliff’s delta to estimate the effect sizes.

For a more meaningful identification of predictor variables for either involuntary hospitalisation at admission or in the course of inpatient treatment, the Chi-square Automatic Interaction Detector (CHAID) was used. CHAID is an algorithm that generates decision trees by performing multiple chi-square tests. It thereby analyses interactions between the different variables which may be continuous or categorical. CHAID can be beneficial due to its ability to visually present a hierarchy of prediction factors. It shows the most significant interactions of variables and thereby indicates the most relevant possible prevention potentials [48]. CHAID became a regularly used method for risk assessments in health research. For example, it was used to predict delirium among patients in medical wards [49], readmission to internal medicine hospital wards [50] and obesity among children [51]. It was also previously used in mental health research, e.g. to predict the success of methadone treatments [52], post-traumatic stress disorders (PTSD) among war veterans [53], and the outcome of vocational rehabilitation for patients with affective disorders [54].

Although CHAID is easy to interpret, its limitations lay in the lack of ensemble techniques like bagging and random split selection. Another limitation is the poor handling of missing data. In order to obtain more robust information, we validated the results using a Random Forest model. The Random Forest algorithm selects random subsets and random variables of the dataset to create multiple decision trees. It thereby avoids overfitting, a common problem with decision tree algorithms, and is relatively robust to outliers and noise [55]. Random Forest models have become increasingly popular lately and have been previously used to predict the suicide risk of medical students [56] and the interaction between cognitive impairment in patients with schizophrenia and psychological distress and the immune system [57].

For the CHAID and Random Forest models, we performed a complete case analysis (*n* = 547) by listwise deletion of all cases with at least one missing value, and, in addition, we analysed all cases (*n* = 1,719) after imputation of missing data based on the proximity data from the Random Forest. As an orienting sensitivity analysis, we compared the two CHAID models by means of AUC and the two Random Forest models by means of AUC and the out-of-bag error rate (OOB) to check for differences in the model performance.

An Exhaustive CHAID on the datasets with and without imputed data was performed, including all clinical, sociodemographic and environmental socioeconomic characteristics. Split-sample validation was used with a random training sample of 70% and a test sample of 30%. The significance level for node splits and combination of categories was set at *p* ≤ 0.05 adapted with the Bonferroni method. The minimum case number for parent nodes was defined as *n* = 50 for the dataset without imputed data and *n* = 100 for the dataset with imputed data. The minimum child node size was defined as *n* = 20 and *n* = 50 respectively. The maximum tree depth was set at 3 levels beneath the root node. To keep the tree diagram lucid, the number of intervals for metric variables was set at 3.

For the Random Forest analysis, we also performed a random 70% training and 30% testing split of both datasets and controlled for the equal distribution of the variables in the training and testing sample. Based on the out-of-bag error rate (OOB), we decided for a tuned Random Forest model with 3 variables randomly sampled as candidates at each split. A total of 500 decision trees were grown and included in the Random Forest model. We measured the relevance of the included variables by the mean decrease in accuracy and mean decrease in Gini, which is a measure of impurity of the nodes. The higher the decrease in Gini the more the variable contributes to high purity in the node splits. The descriptive analysis and the CHAID were carried out with IBM SPSS Statistics version 26 and 27. The Random Forest analysis was carried out with the randomForest package version 4.6–14 in R version 4.0.5.

## Results

### Sociodemographic characteristics

**Fig. 1:**
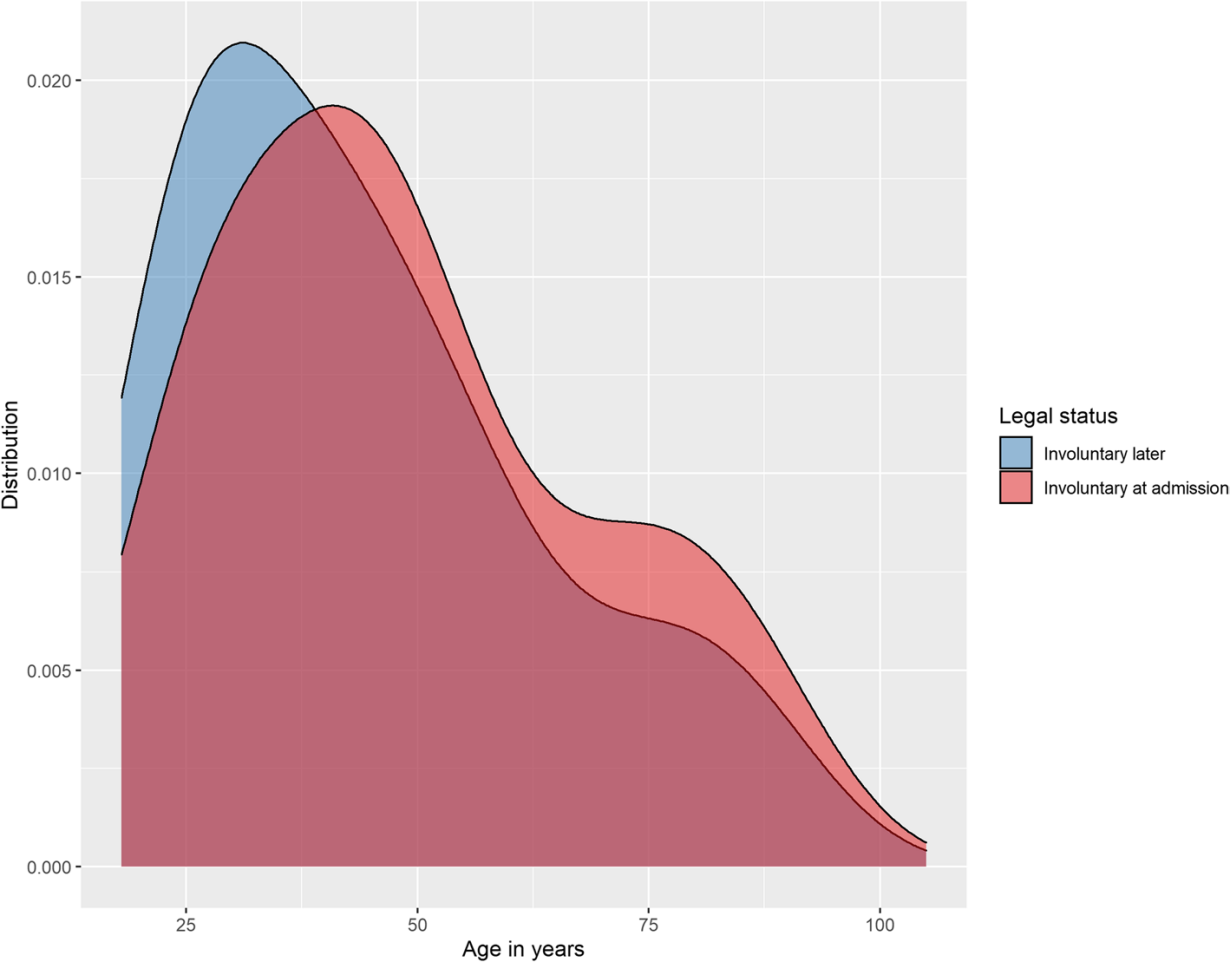
Age distribution depending on legal status

Findings are summarised in Table 1. No significant differences were found between the two groups in regard to gender, marital status, relationship status, living situation, school education, degree of employment, main source of income and existence of children. Significant group differences were found regarding age, migration background, professional education and state of employment, however effect sizes were low. Cases that were involuntarily hospitalised at admission were older both when divided into age groups and as metric variable. For details on the age distribution consider Fig. 1. Also, they were somewhat less likely to have a migration background compared to patients who were involuntarily hospitalised later during their inpatient treatment. Patients who began their inpatient treatment voluntarily but were treated under the Mental Health Act later on were more often unemployed and lacking professional education.

**Table 1:** Sociodemographic characteristics

Category	PsychKG at admission	PsychKG later	Bonferroni*	Missing	Statistical measures	p	Cramér’s V/ Cliff’s delta
**Gender**							
Female	*n *= 604 44.2%	*n* = 165 46.9%		0	χ^2^(1) = .820	*p* = .365	
Male	*n *= 763 55.8%	*n* = 187 53.1%					
**Age**							
Mean	49.03	44.26		0	W = 205,028	*p* < .001	.148 [CI .078-.214]
Standard deviation	19.982	19.554					
**Age (by age group)**							
≤ 40	*n* = 519 38.0%	*n* = 180 51.1%	a	0	χ^2^(2) = 20.828	*p* < .001	.110
41–60	*n* = 481 35.2%	*n* = 104 29.5%	b				
> 60	*n *= 367 26.8%	*n* = 68 19.3%	b				
**Marital status**							
Single	*n* = 690 53.0%	*n* = 190 55.6%		*n *= 74 4.3%	χ^2^(4) = 1.688	*p* = .793	
Married	*n* = 273 21.0%	*n* = 62 18.1%					
Widowed	*n* = 143 11.0%	*n* = 37 10.8%					
Divorced	*n* = 155 11.9%	*n* = 40 11.7%					
Living apart	*n* = 42 3.2%	*n* = 13 3.8%					
**Relationship**							
Yes	*n* = 460 39.0%	*n* = 120 39.9%		*n* = 239 13.9%	χ^2^(1) = .073	*p* = .787	
No	*n* = 719 61.0%	*n* = 181 60.1%					
**Children**							
Yes	*n* = 536 47.6%	*n* = 133 43.3%		*n* = 285 16.6%	χ^2^(1) = 1.741	*p* = .187	
No	*n* = 591 52.4%	*n* = 56,7 56.7%					
**Migration background**							
Yes	*n* = 409 30.1%	*n* = 127 36.1%		*n* = 7 0.4%	χ^2^(1) = 4.690	*p* = .030	.052
No	*n *= 951 69.9%	*n *= 225 63.9%					
**Living situation**							
Alone	*n *= 498 38.8%	*n* = 121 35.5%		*n* = 95 5.5%	χ^2^(4) = 6.748	*p* = .150	
Family/ partner	*n *= 463 36.1%	*n* = 129 37.8%					
Community	*n* = 51 4.0%	*n* = 6 1.8%					
Assisted accommodation	*n* = 188 14.7%	*n *= 61 17.9%					
Emergency accommodation/ homeless	*n* = 83 6.5%	*n* = 24 7.0%					
**School education**							
No graduation	*n *= 134 17.4%	*n* = 43 18.4%		*n* = 713 41.5%	χ^2^(3) = 4.471	*p* = .215	
Lower secondary school	*n* = 256 33.2%	*n* = 71 30.3%					
Higher secondary school	*n* = 159 20.6%	*n* = 62 26.5%					
A-levels	*n* = 223 28.9%	*n* = 58 24.8%					
**Professional education**							
None	*n* = 354 38.1%	*n* = 121 45.5%	a	*n* = 523 30.4%	χ^2^(3) = 12.720	*p* = .005	.103
Apprenticeship	*n* = 353 38.0%	*n* = 104 39.1%	a				
Master apprenticeship	*n* = 102 11.0%	*n* = 12 4.5%	b				
University	*n* = 121 13.0%	*n* = 29 10.9%	a, b				
**Professional situation**							
Employed	*n* = 197 17.1%	*n* = 44 14.7%	a	*n* = 266 15.5%	χ^2^(4) = 11.522	*p* = .021	.089
Unemployed	*n* = 396 34.3%	*n* = 130 43.3%	a				
Homemaker	*n* = 72 6.2%	*n *= 12 4.0%	a				
Retired	*n* = 436 37.8%	*n* = 96 32.0%	a				
In training	*n* = 52 4.5%	*n* = 18 6.0%	a				
**Degree of employment**							
None	*n* = 972 87.8%	*n* = 263 88.3%		*n* = 314 18.3%	χ^2^(2) = 1.461	*p* = .482	
Full time	*n* = 103 9.3%	*n* = 30 10.1%					
Part time	*n* = 32 2.9%	*n* = 5 1.7%					
**Main source of income**							
Employment	*n* = 188 17.9%	*n* = 48 16.9%		*n* = 386 22.5%	χ^2^(4) = 6.234	*p *= .182	
Pension	*n* = 418 39.8%	*n* = 94 33.1%					
Own assets	*n* = 5 0.5%	*n* = 2 0.7%					
Unemployment benefits	*n* = 374 35.7%	*n* = 117 41.2%					
Alimony	*n* = 64 6.1%	*n* = 23 8.1%					

^*^ Each letter denotes a subset whose column proportions do not differ significantly from each other at the .05 level

### Environmental socioeconomic characteristics

Findings are summarised in Table 2. There were no group differences in seven out of eight variables. Significant differences were found for the number of children per 100 inhabitants in the neighbourhood. Cases with PsychKG in the course of inpatient treatment came from areas with a denser children population. However, the effect size of this finding was small. 9.8% of the cases could not be linked with environmental sociodemographic characteristics, mostly because of patients without a permanent place of residence.

**Table 2:** Environmental socioeconomic characteristics

Category	PsychKG at admission	PsychKG later	Statistical measures	p	Cliff’s delta (95% CI)
*n* = 1,550 Missing: 9.8%	*n *= 1,233	*n *= 317			
**Commercial enterprises per 100 inhabitants**					
Mean	8.60	7.95	W = 188,838	*p* = .353	
Standard deviation	5.70	3.69			
**Unemployment per 100 inhabitants**					
Mean	7.46	7.25	W = 190,426	*p* = .481	-
Standard deviation	3.10	2.92			
**Employment per 100 inhabitants**					
Mean	68.91	68.91	W = 198,916	*p* = .624	-
Standard deviation	3.00	2.77			
**Buildings per 100 inhabitants**					
Mean	14.53	15.08	W = 207,447	*p* = .091	-
Standard deviation	5.09	5.13			
**Residential buildings per 100 inhabitants**					
Mean	14.35	14.89	W = 206,525	*p* = .118	-
Standard deviation	5.05	5.11			
**Households per 100 inhabitants**					
Mean	53.11	52.57	W = 192,338	*p* = .663	-
Standard deviation	6.11	5.53			
**Children per 100 inhabitants**					
Mean	13.11	13.25	W = 215,873	*p* = .004	.103 (CI .033-.173]
Standard deviation	0.57	0.67			
**Purchasing power per 100 inhabitants (in Euro)**					
Mean	2,194,560	2,194,56	W = 204,413	*p* = .206	-
Standard deviation	314,927	276,442			

### Clinical and systemic characteristics

All details are shown in Table 3. In terms of the main diagnosis, patients with an organic mental disorder (ICD-10: F0) were more often involuntarily hospitalised at admission. Also, cases with mental and behavioural disorders due to psychoactive substance use (ICD-10: F1) were overrepresented in that group. Patients with schizophrenia, schizotypal and delusional disorders (ICD-10: F2) on the other hand were overrepresented among the cases that were detained during the later course of inpatient treatment. The group difference was significant, but effect size was low. Group differences were also significant when including both the main and all secondary diagnoses in the analysis, but, again, effect sizes were very low. 21.7 vs. 14.2% of the cases were diagnosed with an organic mental disorder, 49.3 vs. 41.5% with a substance abuse disorder and 31.2% vs. 46.6% with a psychotic disorder. Behavioural and emotional disorders with onset usually occurring in childhood and adolescence (ICD-10: F9) were also overrepresented among patients treated under the Mental Health Act in the course of their hospitalisation, but only with *n* = 6 (0.4%) vs. *n* = 5 (1.7%) cases.

**Table 3:** Clinical and systemic characteristics

Category	PsychKG at admission	PsychKG later	Bonferroni*	Missing [%]	Statistical measures	p	Cramér’s V/ Cliff’s delta
**Main diagnosis (ICD-10)**							
F0	*n* = 270 19.8%	*n* = 47 13.4%	a	0	χ^2^(6) = 36.983	*p* < .001	.147
F1	*n* = 341 24.9%	*n* = 66 18.8%	a				
F2	*n* = 387 28.3%	*n *= 146 41.5%	b, c				
F3	*n* = 224 16.4%	*n* = 56 15.9%	a, b, c				
F4	*n* = 76 5.6%	*n* = 11 3.1%	a, c				
F6	*n *= 49 3.6%	*n* = 13 3.7%	a, b, c				
Other	*n* = 20 1.5%	*n* = 13 3.7%	b				
**Main or secondary diagnoses (ICD-10)**							
F0	*n* = 296 21.7%	*n* = 50 14.2%		0	χ^2^(1) = 9.661	*p* = .002	.075
F1	*n* = 674 49.3%	*n* = 146 41.5%		0	χ^2^(1) = 6.875	*p* = .009	.063
F2	*n* = 426 31.2%	*n* = 164 46.6%		0	χ^2^(1) = 29.556	*p* < .001	.131
F3	*n* = 308 22.5%	*n* = 82 23.3%		0	χ^2^(1) = .093	*p* = .760	
F4	*n* = 137 10.0%	*n* = 27 7.7%		0	χ^2^(1) = 1.793	*p* = .181	
F6	*n* = 180 13.2%	*n* = 57 16.2%		0	χ^2^(1) = 2.156	*p* = .142	
F7	*n* = 15 1.1%	*n* = 7 2.0%		0	χ^2^(1) = 1.760	*p* = .185	
F9	*n* = 5 0.4%	*n* = 6 1.7%		0	χ^2^(1) = 7.891	*p* = .005	.068
**Dual diagnoses (comorbidities)**							
F1 + F2	*n* = 153 11.2%	*n* = 50 14.2%		0	χ^2^(1) = 2.439	*p* = .118	
F1 + F6	*n* = 122 8.9%	*n* = 31 8.8%		0	χ^2^(1) = .005	*p* = .945	
**Suicidal tendencies upon admission**							
Yes	*n* = 559 41.2%	*n *= 93 26.6%		*n* = 14 0.8%	χ^2^(1) = 24.927	*p* < .001	.121
No	*n* = 797 58.8%	*n* = 256 73.4%					
**Previously attempted suicide(s)**							
Yes	*n* = 288 29.8%	*n* = 77 29.8%		*n* = 496 28.9%	χ^2^(1) = .000	*p* = 1	
No	*n* = 677 70.2%	*n* = 181 70.2%					
**Treatment prior to admission**							
No previous treatment	*n *= 572 41.8%	*n* = 105 29.8%		0	χ^2^(1) = 16.924	*p* < .001	.099
Previous outpatient treatment	*n* = 407 29.8%	*n* = 124 35.2%		0	χ^2^(1) = 3.900	*p* = .048	.048
Contact to socio-psychiatric services	*n* = 41 3.0%	*n* = 1 0.3%		0	χ^2^(1) = 8.658	*p* = .003	.071
Day-care hospital	*n* = 187 13.7%	*n* = 96 27.3%		0	χ^2^(1) = 37.608	*p* < .001	.148
**Previous psychiatric inpatient treatment(s)**							
Yes	*n* = 855 68.1%	*n* = 275 82.1%		*n* = 128 7.4%	χ^2^(1) = 25.246	*p* < .001	.126
No	*n* = 401 31.9%	*n* = 60 17.9%					
**Treating hospital**							
Hospital 1	*n* = 959 70.2%	*n* = 190 54.0%	a	0	χ^2^(3) = 92.277	*p* < .001	.232
Hospital 2	*n* = 158 11.6%	*n* = 114 32.4%	**b**				
Hospital 3	*n* = 150 11.0%	*n* = 33 9.4%	**a**				
Hospital 4	*n* = 100 7.3%	*n* = 15 4.3%	**a**				
**Time of admission**							
Regular service hours	*n* = 499 36.5%	*n* = 133 37.8%		0	χ^2^(1) = .198	*p* = .657	
Outside service hours	*n* = 868 63.5%	*n* = 219 62.2%					
**Length of inpatient stay**							
Mean	24.69	36.78		0	W = 302,726	*p* < .001	.258 [CI .190-.320]
Standard deviation	34.583	38.151					

^*^ Each letter denotes a subset whose column proportions do not differ significantly from each other at the .05 level

Cases with suicidal tendencies upon admission were rather immediately involuntarily hospitalised than later during their treatment. Furthermore, patients who received no outpatient treatment prior to their inpatient stay were more likely to be involuntarily hospitalised at admission. Patients on the other hand who received either outpatient treatment or treatment in a day-care hospital and cases who had a history of previous psychiatric inpatient treatment were more often detained later during their hospitalisation. Again, all effect sizes were small.

Interestingly, the allocation to the treating hospital made a significant difference, with hospital 2 having a higher proportion of detentions during the course of inpatient stay compared to hospitals 1, 3 and 4. Admission within or outside regular service hours made no difference. Cases that had been in touch with the municipal socio-psychiatric services prior to admission were more often confined at admission. Finally, patients who were detained during the course of inpatient treatment stayed significantly longer hospitalised than involuntarily admitted cases.

### Chi-squared Automatic Interaction Detection (CHAID)

**Fig. 2:**
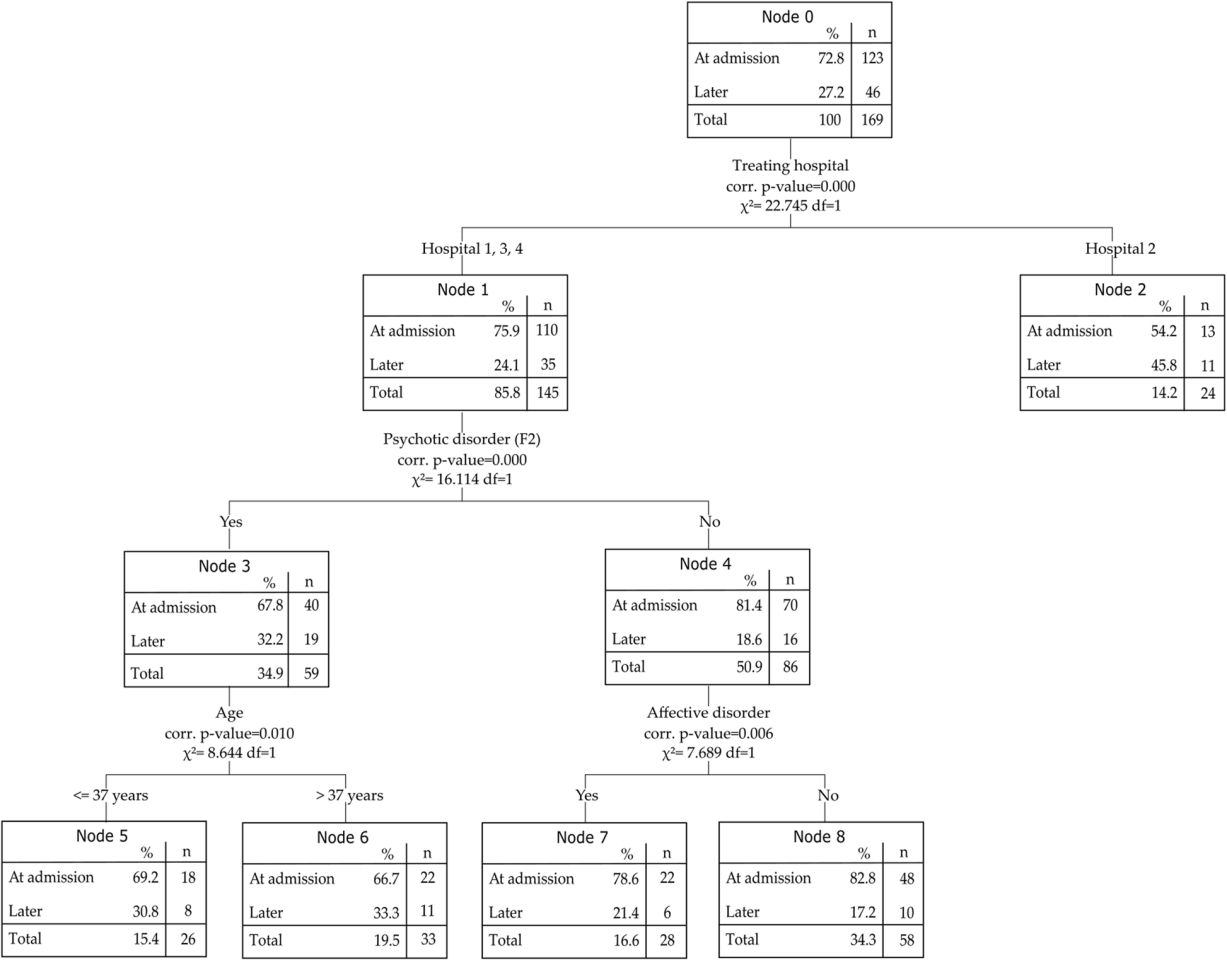
CHAID decision tree model on the cases without missing data (complete case analysis)

The Exhaustive CHAID analysis of the complete case dataset led to 4 included variables represented in 9 nodes (Fig. 2). 72.8% (95% CI = 66.1, 79.5) of the cases were correctly predicted in the test sample but in fact the model predicted 100% of the cases to be involuntary at admission. The area under the curve for the test sample was AUC = 0.703.

**Fig. 3:**
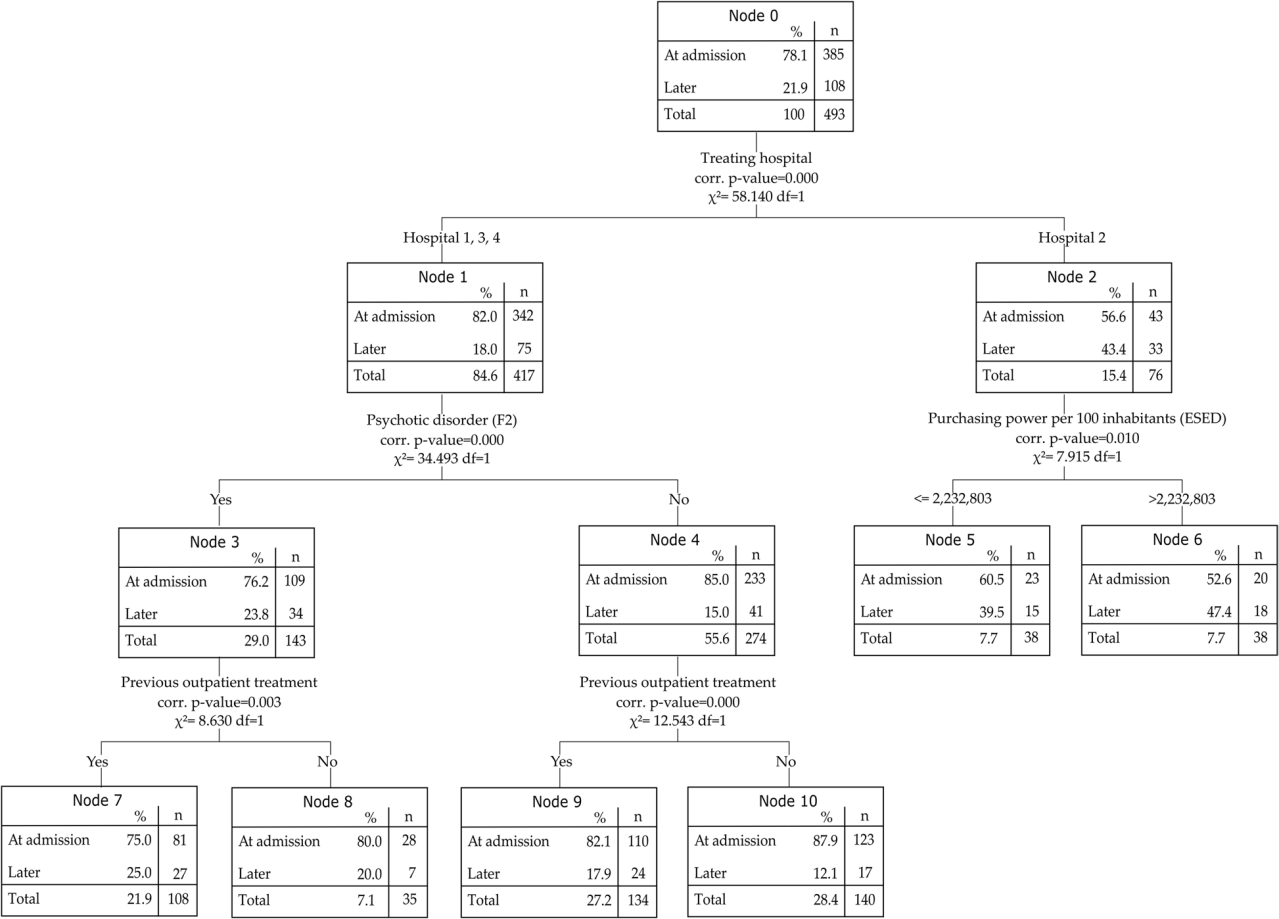
CHAID decision tree model on the imputed dataset

The CHAID analysis on the imputed dataset equally consisted of 4 included variables and 11 nodes (Fig. 3). 76.5% (95% CI = 72.8, 80.2) of the cases were correctly predicted. The model performed better in correctly predicting the cases with PsychKG at admission. For the outcome of involuntary hospitalisation later during treatment the sensitivity was 13.9% and the specificity 94.0% which equals to a balanced accuracy of 54.0%. The AUC was 0.693 indicating a similar model fit compared to the complete case analysis.

In the CHAID model based on the complete case dataset, the first split was based on the treating hospital. Cases that were treated in hospital 2 more often experienced a change of legal status. For the cases in hospital 1, 3 and 4, the second split of the model was based on the presence of a psychotic disorder. Cases without a psychotic disorder were detained more often directly at admission. The cases without a psychotic disorder (node 4) were further differentiated by the presence of an affective disorder. The cases with an affective disorder were less often detained at admission than those without. Among the cases with a psychotic disorder (node 3), cases up to the age of 37 were more often involuntarily hospitalised at admission.

In the CHAID model on the imputed dataset, the first split was also based on the treating hospital and the cases in hospital 1,3 and 4 were again split based on presence of a psychotic disorder (node 3 and 4). Cases with as well as without a psychotic disorder that previously received outpatient treatment were less likely to receive involuntary treatment since admission (node 7 vs 8 and node 9 vs 10). The cases in hospital 2 were further divided based on the purchasing power per 100 inhabitants. Cases from an area with lower purchasing power were more often detained at admission.

### Random Forest

The Random Forest model based on the imputed dataset predicted 80.9% (95% CI = 77.2, 84.2) correctly when applied to the test sample. The sensitivity was 10.2% and specificity 97.6% which results in a balanced accuracy of 53.9%. The area under the curve of AUC = 0.712 was slightly higher than for the CHAID model. The out-of-bag error rate (OOB) was 20.8%. To ensure that variables with a high proportion of missing values did not distort the results of the model, we computed the Random Forest model again after exclusion of the variable *school education*, which had the highest proportion of missing values (41.5%). The model fit was very similar to the previous computation with an AUC of 0.710.

**Fig. 4:**
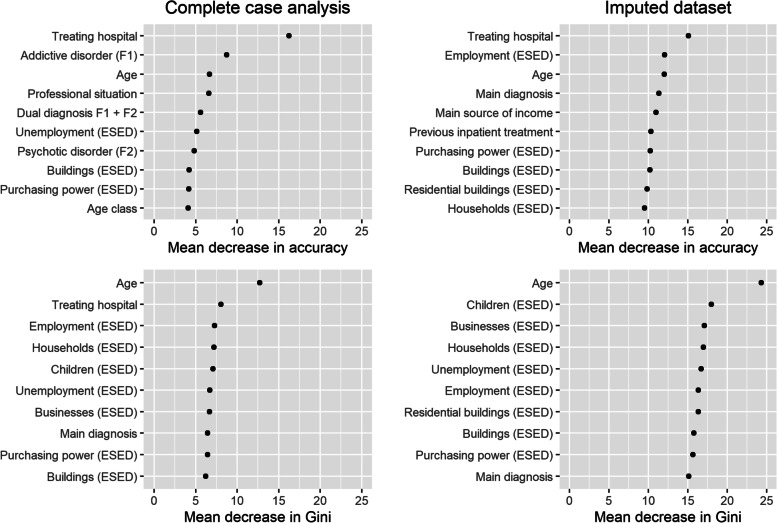
Random Forest model – The ten highest mean decreases in accuracy and Gini (impurity of the splits)

The complete case Random Forest model had an OOB of 28.8% and AUC = 0.756 which indicates no bigger signs of distortion in comparison to the imputed dataset model. The Random Forest model on the complete case dataset achieved an accuracy of 82.9% (95% CI = 76.1, 88.4). The sensitivity was 28.1% and the specificity 96.8% which equals a balanced accuracy of 62.4%. The variables that caused the highest decrease in Gini and accuracy both for the complete case and imputed dataset model are shown in Fig. 4.

The most important variables for the accuracy of the imputed dataset model were determined as the treating hospital, age, the main diagnosis, main source of income and previous inpatient treatment. Several ESED were identified as important variables for the accuracy of the model as well. Age and the main diagnosis were also identified to have an impact on the purity of the node splits (Gini). Again, the ESED played an important role. The complete case analysis yielded similar results with addictive and psychotic disorders playing a bigger role in the model.

## Discussion

The present study included the data of the 1,773 cases who were treated under the PsychKG NRW (Mental Health Act). The majority of clinical, systemic, sociodemographic and environmental socioeconomic characteristics did not differ between those who were admitted against their will (*n* = 1,367) and those who were admitted initially on their own will and were confined according to the Mental Health Act at some later point during in-patient treatment (*n* = 352). Cases that were admitted against their will were on average about five years older, they had somehow less often a migration background, had a slightly better professional education and were less often unemployed compared to those who were admitted initially on their own will and were confined according to the Mental Health Act at some later point during in-patient treatment. They lived in areas with fewer children per 100 inhabitants. They were less often diagnosed with a psychotic disorder and more often with an organic mental disorder or a substance abuse disorder. Unsurprisingly, they also showed higher suicidal tendencies upon admission, and they received less often outpatient or day patient treatment prior to admission. Also, they had less often a history of psychiatric inpatient treatments in the past. Finally, the average length of inpatient treatment for these cases was shorter. This latter finding may be interpreted as an impact of an extended treatment duration in cases with switch from voluntary to involuntary status due to worsened symptoms in the course of treatment. However, all effect sizes were small and the machine learning algorithms did not yield a causally interpretable prediction model: Both the CHAID and Random Forest models achieved unsatisfying accuracy rates, especially in the correct prediction of cases with switch of legal status from voluntary to involuntary during the course of inpatient stay. Another indicator for the limited explanatory power of the analysed variables is the small differences in decrease of accuracy and Gini per variable in the Random Forest model. None of the observed group differences in the descriptive analyses played a sufficient role in the tree models to be identified as an important determinant with reasonable certainty.

In summary, although we included a large number of socioeconomic, demographic, clinical and systemic variables, the characteristics were not sufficient to draw a clear differentiation between the two groups. The small differences we found are not easy to interpret, but it appears that also local differences in the handling of patients at risk of self-harm or harm to others and/ or differences in the patient populations of the hospitals play a role, as the treating hospital was one of the predominant influencing factors in the CHAID analysis. In an explorative post-hoc analysis, we investigated possible differences between the cases from hospital 2 and the other hospitals. Cases in hospital 2 were significantly older and more often retired. The main diagnosis in hospital 2 was more often an organic mental disorder and the cases less often showed suicidal tendencies at admission. Furthermore, also the ESED differed significantly: We found a higher purchasing power and lower unemployment rate per 100 inhabitants in the living environment of cases from hospital 2. Nonetheless, the reasons for these differences are subject to speculation and cannot be dissolved based on the data presented.

In conclusion, our results imply that there are more similarities than differences between the two subgroups of patients. Hence, it seems justified to consider them as one group. This applies both to further research activities and to planning preventive measures against coercion.

### Strengths and limitations

The present study is based on a detailed in-depth analysis of health records of a comparatively large sample as we were able to include all cases of inpatient treatment under the Mental Health Act of an entire year and all four psychiatric hospitals of the Metropolitan city of Cologne, Germany. The districts the psychiatric hospitals provide care for are different in terms of socioeconomic characteristics and the procedures of involuntary hospitalisation might differ from hospital to hospital; however, by investigating all cases, we minimised distortion. Also, we were able to minimise distortion due to other systemic factors, as a single municipal court decides on all involuntary hospitalisations in the city of Cologne. Furthermore, other communal support structures, such as the socio-psychiatric services and outpatient treatment facilities, are comparable throughout the different sectors of the city. Another strength of our study is the application of two machine learning algorithms, the exhaustive CHAID and Random Forest. CHAID can increase the lucidity of the results and help depicting interactions between variables [48]. The additional usage of the Random Forest algorithm promised a further improvement of the accuracy of the findings [55].

A limitation of the study is the high number of missing values for some variables such as school education, professional education, main source of income and previously attempted suicides. We addressed this issue by comparing complete case models with imputation-based models. The analysis of the model robustness indicated no stronger influence of missing data patterns. Furthermore, the clinical records analysed in this study lacked other potentially interesting information such as adherence to pharmacological treatment at admission, symptom severity and social functioning level based on assessments using standardised psychometric instruments, previous psychoeducation, level of insight, and perceived social and professional support. These limitations are pertinent to the retrospective nature of the analysis. A prospective study design would have the potential to ensure more reliable results on the factors with many missing items and could detect more determinants that play a role in the occurrence of involuntary hospitalisation in general and a switch of voluntariness status during treatment in particular.

Finally, it is not clear how far our findings can be generalized. The legal framework and the practical procedures on the application of Mental Health Act in general and the switch of legal status during inpatient treatment seem to differ grossly in an international perspective. While our results are probably comparable to the situation in other metropolitan regions of Germany, this aspect of involuntary hospitalisation might be less prevalent in other regions and countries. In our sample, cases with a change of voluntariness status accounted for about one fifth of all cases with detention under the Mental Health Act. In comparison, a study from Canada reported *n* = 676 of such cases compared to a total number of 250,773 observed cases before exclusion which makes up less than 1% of the entire sample [37]. Also, a study from Brazil mentions the possibility of a converted voluntariness status in the course of treatment in Brazil but refers to it as something that in practice “seldom occurs” [58]. They nonetheless did not clarify whether or not such cases were included.

We suggest that both differences in the legal frameworks of involuntary hospitalisation and in local working practices and procedures might account for the variabilities found. The latter is in line with our finding of differences in the proportion of Mental Health Act cases with a switch in legal status among the four hospitals included in our study.

## Conclusions

Despite some group differences, we were not able to establish a reliable model on determinants of compulsory hospitalisation under the Mental Health Act right at admission vs. later on during the course of inpatient treatment. The two groups seem to be more alike than different. We therefore propose to include cases with a switch of legal status from voluntary to involuntary during inpatient treatment in further research in this field.

## Data Availability

The dataset used for this study contains all involuntarily treated cases of an entire year of four hospitals and includes the number of cases per hospital as well as the respective length of inpatient stay. As it is thereby possible to draw conclusions on the economic situation of the hospitals, the dataset used and analysed during the current study is only available from the corresponding author on reasonable request.

## Abbreviations

AUC: Area under the curve
BGB: Civil Law Code
CHAID: Chi-squared Automatic Interaction Detection
ESED: Environmental socioeconomic data
OOB: Out-of-bag error rate
PsychKG: Mental Health Act
